# Electrolyte-driven modulation of charge storage mechanisms in Co metal–organic frameworks for advanced supercapacitors

**DOI:** 10.1039/d6ra01795a

**Published:** 2026-04-15

**Authors:** Mrinalini Sharma, Manas Nasit, Nitin Kumar Gautam, Shruti Lavania, Saurabh Dalela, P. A. Alvi, Nagih M. Shalaan, Ranjeet Kumar Brajpuriya, Aditya Sharma, Shalendra Kumar

**Affiliations:** a Department of Physics, School of Advanced Engineering, UPES Dehradun 248007 India ranjeet.brajpuriya@ddn.upes.ac.in shailuphy@gmail.com; b Department of Pure & Applied Physics, University of Kota Kota Rajasthan 324005 India; c Department of Physical Science, Banasthali Vidyapith Banasthali Rajasthan 304022 India; d Department of Physics, College of Science, King Faisal University P. O. Box 400 Al-Ahsa 31982 Saudi Arabia

## Abstract

This study examines the influence of electrolytes and the molarity-dependent electrochemical evaluation of Co-MOF-based electrodes for supercapacitor applications. The synthesized Co-MOF was analyzed using XRD, FTIR and FE-SEM techniques, which collectively confirmed the successful formation of the material. The electrochemical performance was evaluated using CV, GCD and EIS in alkaline electrolytes of different molarities. Co-MOF exhibited a *C*_sp_ of 379.31 F g^−1^ and 852.5 F g^−1^ in KOH and NaOH, respectively, at a scan rate of 2 mV s^−1^, indicating superior response in NaOH. Similarly, GCD measurements revealed an enhanced *C*_sp_ of 1147.2 F g^−1^ at 0.5 A g^−1^ in NaOH, compared with 317.86 F g^−1^ in KOH. The molarity of the electrolyte was varied (1 M NaOH, 3 M NaOH and 5 M NaOH), and 1 M NaOH displayed optimal performance, while maintaining a ∼98% of the capacitance retention after 10 000 cycles. A symmetric Co-MOF Swagelok supercapacitor utilizing 1 M NaOH showed a *C*_sp_ of 37.7 F g^−1^ (CV) and 14.9 F g^−1^ (GCD) at 0.25 A g^−1^ with a maximum *E*_d_ of 3.73 W h kg^−1^ at a *P*_d_ of 118.75 W kg^−1^ and ∼43.96% retention after 10 000 cycles. Similarly, at a scan rate of 2 mV s^−1^, the Co-MOF pouch cell exhibited a *C*_sp_ value of 21.42 F g^−1^ from CV and a peak capacitance of 1.68 F g^−1^ when evaluated at 0.25 A g^−1^ for GCD. Ragone analysis revealed that the device delivered an *E*_d_ of 0.22 W h kg^−1^ at a corresponding *P*_d_ measured at 43.75 W kg^−1^. The results underscore the importance of concentration and electrolyte selection as critical parameters for Co-MOF supercapacitor performance.

## Abstract 2

This study examines the influence of electrolytes and the molarity-dependent electrochemical evaluation of Co-MOF-based electrodes for supercapacitor applications.

## Graphical abstract

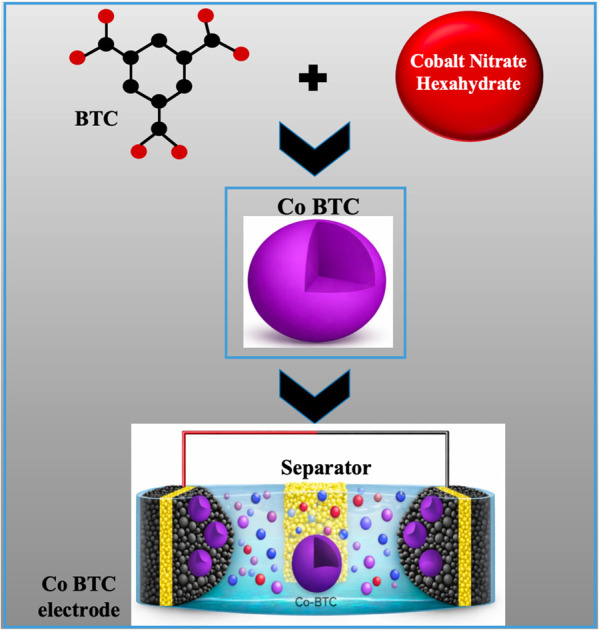

## 1. Introduction

Since the Industrial Revolution, fossil fuels have long been the main source of energy, supporting economic development. However, their extensive use has caused significant global environmental problems, including air pollution, the greenhouse effect, and photochemical smog.^1–4^ As the global population rises, the energy demand is expected to increase, potentially triggering an energy crisis.^5,6^ As a result, supercapacitors are gaining significant research interest for energy storage because of their excellent rate capability, delivering high power densities upto 10 kW kg^−1^, and prolonged operational lifespans.^7–9^ In contrast to batteries, they display higher specific capacitance (*C*_sp_) and simultaneously lower internal resistance. Supercapacitors offer considerable potential for advanced applications, including fuel cells and electric vehicles, and are also widely employed in medical devices and consumer electronics.^10–13^ Based on the storage mechanism, supercapacitors are primarily categorized into two main types: electric double-layer capacitors (EDLCs) and pseudocapacitors. In EDLCs, charge is stored by the electrostatic accumulation of ions at the interface between the electrode and electrolyte, and these devices are mainly composed of carbon-based materials, such as carbon nanotubes (CNTs) and activated carbon (AC), which provide a high surface area for charge storage.^14,15^ In contrast, pseudocapacitors involve reversible redox reactions and commonly utilize materials such as conductive polymers, metal sulfides/oxides/phosphides, and metal hydroxides.^16,17^ Due to the faradaic process, pseudo-capacitors exhibit higher capacitance, while EDLC materials offer greater conductivity and stability.^18–20^ Current research efforts are focused on achieving high energy density in supercapacitors, with two main areas: synthesizing electrode materials with higher surface area, and developing newer electrode materials that offer higher capacitance and a broader potential window.

Over the past few years, researchers have tested several materials for their suitability in supercapacitors, including graphene, metal oxides, CNTs, MXenes, carbon nitride, and metal–organic frameworks (MOFs).^21–27^ Among these, MOFs have received considerable research interest due to their extensive large surface area, ordered crystalline frameworks, tunable pore dimensions and chemically versatile structures.^28,29^ They are comprised of metal ions bridged by organic ligands (linkers), forming well-defined one-, two-, or three-dimensional networks. In addition, their abundance of active sites enhances ion transport and redox activity, making MOFs highly suitable for boosting charge storage and improving the overall performance of supercapacitor devices.^30,31^

MOFs can be utilized for electrode fabrication in supercapacitor devices by two distinct methods. Firstly, Pristine MOFs are capable of storing electrical energy on their internal surfaces, exhibiting both bi-layer and pseudocapacitive behaviour arising from redox-active metal sites, and on the other hand, can be employed as precursors to synthesize diverse functional products, such as metal oxides, metal-based hydroxides, composite metal oxides, metal hydroxide compounds, porous carbon compounds and sulfides.^32–34^ Pristine MOFs are viable candidates for supercapacitor electrode materials and are also utilized directly as electrode materials, exhibiting pseudocapacitive characteristics owing to their metal centers. However, one significant drawback of these materials is their low conductivity and limited ion transport.^32^ These drawbacks can be overcome by pairing the right metal centers with organic linkers to enhance the overall framework.^30^ Recent advances in MOFs, where metal nodes are coupled with π-conjugated linkers, have significantly enhanced charge transport within the structure.^35,36^ Organic linkers are especially important as they have tunable porosity, redox activity, and ion diffusion.^37^ For instance, trimesic acid, also known as 1,3,5-benzenetricarboxylic acid, with its three carboxyl groups, enables the construction of multidimensional frameworks through hydrogen bonds, coordination bonds, and π–π interactions. By adjusting their linker chemistry, MOFs can be engineered to feature shorter diffusion pathways and abundant electroactive sites, thereby improving their electrochemical behavior.^38–40^

Many studies have highlighted the efficiency of MOFs in supercapacitor applications, with specific emphasis on those involving Ni, Zn, Cu, Mn, Cd, and Fe.^41–46^ Recently, Co-based MOFs have been gaining attention among researchers owing to their higher capacities and sufficient structure stability during repeated cycles.^47^ Co provides abundant active sites, exhibits multivalency and excellent redox properties, facilitating electron transfer during the conversion reaction.^47,48^Co-MOFs possess a large surface area and strong structural stability, facilitating the effective movement of ions and electron transfer through the charge–discharge process.^49,50^ For instance, in 2018, Zhu *et al.* developed a Co-MOF supported on nickel foam as an electrode, which achieved a substantial areal capacitance, reaching 13.6 F cm^−2^ when evaluated with 2 M KOH, and retained 79.4% of its capacitance after the current density (*I*_d_) was elevated to 20 mA cm^−2^.^51^ Recently, in 2025, Snowlin *et al.* reported a Co-based MOF synthesized *via* the solvothermal method, exhibiting high *C*_sp_ and long-term cycling stability, even over 5000 cycles (92.15% retention).^52^ Despite these studies, the electrochemical efficiency of the Co-based MOF remains limited due to its low electrical conductivity and single energy storage mechanism. Interestingly, the MOF performance depends not only on the material itself but also on several factors, including temperature, pressure, and electrolytic stability.^53,54^ Among these, the molarity of the electrolyte profoundly affects the performance of the MOF, enhancing the charge storage mechanism and thereby improving capacitive performance.^55,56^ As such, several factors must be considered, such as conductivity, stability under consistent operating conditions, ion mobility, and viscosity, since each electrolyte offers its own advantages and limitations. Among the most commonly used liquid electrolytes, organic and ionic electrolytes offer a broader working potential window, resulting in higher energy density.^57,58^ Despite these advantages, their inherently low electrical conductivity and high viscosity yield moderate capacitance and sub-optimal electrochemical performance. In contrast, aqueous electrolytes are cost-effective and environmentally benign, offering high ionic conductivity, low viscosity and efficient charge transport with superior performance.^59,60^ Therefore, several studies have examined the impact of various electrolyte on the performance of Co-MOF's, with the goal of improving the storage capacity of the material. In 2018, Xuan *et al.* evaluated the capacitive storage characteristics of Co-MOF in 3 M KOH, demonstrating excellent redox behavior and delivering the highest *C*_sp_ value, reaching more than 950 F g^−1^ when tested at a current density of 0.5 A g^−1^.^61^ In 2023, Gurav *et al.* reported that Co-MOF's/FSS, when tested in a 2 M LiOH electrolyte, delivered a *C*_sp_ value reaching 650 F g^−1^. The electrode also maintained 71% of its initial performance over 5000 GCD cycles at an *I*_d_ of 10 mA cm^−2^.^62^ Despite these advancements, a systematic understanding of the effect of electrolyte concentration on *C*_sp_ remains limited for Co-based MOFs. In addition, reports of practical device fabrication for Co-based MOF remain notably scarce.

Herein, we focus on examining Co-MOF's as electrode materials with an emphasis on understanding how the electrolyte and molarity impact supercapacitor performance. The electrochemical performance of Co-MOF was systematically evaluated using CV, GCD, and EIS analyses in alkaline electrolytes (NaOH and KOH) at different molarities (1, 3 and 5 M). This enabled the investigation of electrolyte concentration on supercapacitor behavior and stability, facilitating the identification of optimal operating conditions. The practical applicability of the synthesized material was assessed *via* the fabrication of a symmetric supercapacitor device, including a Swagelok-type device and a pouch cell configuration. Overall, in this study, we aim to bridge the gap between electrolyte concentration, material performance and device implementation of Co-based MOFs for supercapacitor application.

## 2. Experimental details

### 2.1 Materials

All chemicals used in this study were of analytical grade and employed without any further purification. Cobalt nitrate hexahydrate (Co(NO_3_)_2_·6H_2_O) (Molychem (99% purity)) and 1,3,5-benzenetricarboxylic acid (BTC) (SRL (98% purity)) were utilized as precursor materials for the synthesis. Dimethylformamide (DMF) (SRL (99.9% purity)), along with ethanol and deionized water, served as solvents. For electrode fabrication, polyvinylidene fluoride (PVDF) and AC were used as binder and conductive material, respectively, followed by electrochemical analysis.^63–65^

### 2.2 Synthesis procedure for Co-MOF

**Fig. 1:**
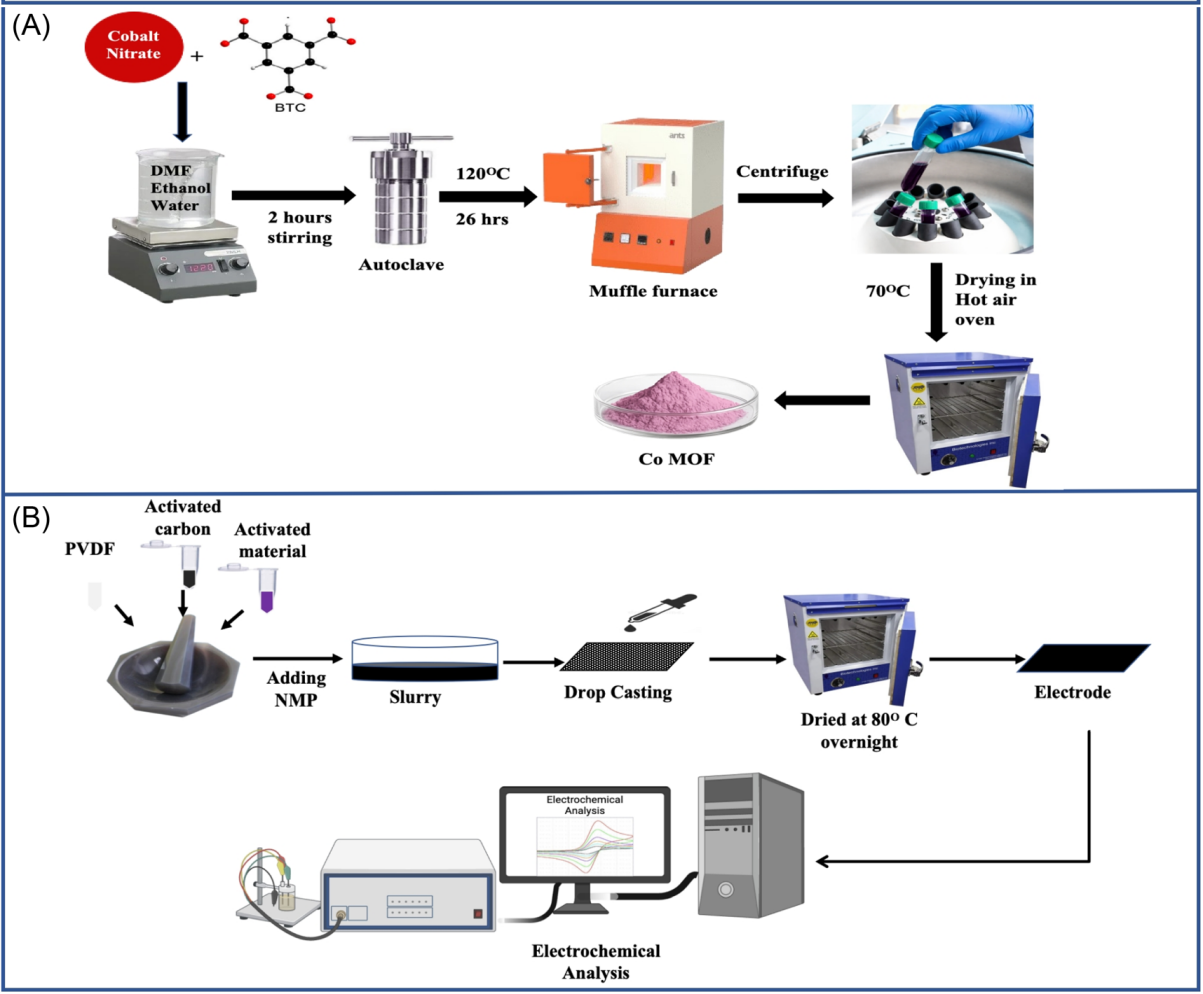
(A) Illustration of the hydrothermal synthesis of Co-MOF. (B) Schematic of the fabrication of the Co-MOF electrode for electrochemical analysis.

The Co-based MOF was prepared using the hydrothermal method, with BTC serving as the organic linker. Initially, 4.8 g of (Co(NO_3_)_2_·6H_2_O) was dissolved in a total volume of 80 mL of DMF, ethanol and water, and 1.2 g of BTC as an organic linker was then carefully introduced into the mixture. After stirring the solution for 2 h to ensure homogeneity, it was kept in a PTFE-stainless steel hydrothermal autoclave and heated at 120 °C for 26 h under hydrothermal conditions. Thereafter, the resulting solution was centrifuged and repeatedly washed with ethanol and DMF to remove any residual reactants or solvents. The obtained solid product was dried at 70 °C in a hot air oven overnight, leading to the synthesis of Co-MOF. The preparation procedure of the Co-MOF is presented in Fig. 1(a).

### 2.3 The method for preparing the working electrode

The electrochemical behaviour of Co-MOF was investigated using a standard three-electrode configuration in 1 M KOH and 1 M NaOH aqueous electrolyte solutions and was tested in 3 M and 5 M NaOH solutions. To prepare the working electrode, 8 mg of the synthesized material was combined with 1 g of AC serving as the conductive additive and 1 g of PVDF as the binder in the ratio of 8 : 1 : 1.^66–68^ This mixture was carefully ground in a mortar by adding 3 to 4 drops of *N*-methyl pyrrolidone (NMP) to obtain a homogeneous suspension. Thereafter, the homogenized paste was deposited onto a nickel foam substrate with dimensions of 2 cm × 1 cm and a mass loading of 1 mg, then kept at 80 °C in a hot air oven for overnight, as presented in Fig. 1(b).

### 2.4 Electrode fabrication for a symmetric supercapacitor (Swagelok cell)

The electrode was fabricated using a simple drop-casting approach, with the active material mixed with the PVDF binder and AC in an 80 : 10 : 10 mass ratio to enhance the mechanical stability and facilitate efficient charge transport. Afterwards, NMP solvent was incorporated into the mixture and stirred thoroughly to obtain a homogeneous slurry. Afterwards, the prepared slurry was carefully drop cast on a circular piece of graphitic paper current collector with a total mass loading of 2 mg to fabricate the electrode. Following this, it was dried at 80 °C in a hot air oven for overnight to remove the residual solvent. Finally, the device was assembled using a Swagelok-type cell with a Whatman filter paper serving as a separator. Prior to assembly, the separator was soaked in 1 M NaOH aqueous electrolyte. The assembled device was denoted as the Co-MOF symmetric supercapacitor.

### 2.5 Electrode fabrication for symmetric supercapacitor (pouch cell)

The fabricated pouch cell electrode was assembled following the same approach; however, in this case, the slurry was applied to a rectangular graphitic paper. A polyvinyl NaOH (PVA–NaOH) gel electrolyte was employed as both a gel electrolyte and a separator. For gel electrolyte preparation, 4.5 g of PVA and 4.5 g of NaOH were introduced into 40 mL of DI water while being continuously stirred at 80 °C for 9 h to facilitate the dissolution of PVA. The uniform solution was carefully placed in a Petridish and the amount was regulated to produce a separator of the required thickness. The cast solution was kept under ambient conditions overnight, resulting in the formation of a solidified gel electrolyte.

For the fabrication of a pouch cell, gel electrolyte served as a separator and was placed between the two electrodes to form the Co-MOF pouch cell with dimensions of 2.5 cm × 1.5 cm, maintaining a total mass loading of 4 mg as shown in Fig. S1, which was then electrochemically evaluated using a two-electrode setup through CV, GCD and EIS analysis.

### 2.6 Characterization of Co-MOF

Powder X-ray diffraction (XRD) analysis was carried out to investigate the sample crystal structure using a Bruker D8 Advance EcoPro diffractometer equipped with copper Kα radiation (*λ* = 1.5406 Å). To identify the chemical bonds and functional groups present in the materials, Fourier transform infrared spectroscopy (FTIR) measurements were carried out using a PerkinElmer spectrometer. The surface chemical composition and elemental oxidation states were determined through X-ray photoelectron spectroscopy (XPS) using a Kratos Analytical AXIS SUPRA^+^ with a monochromatic Al Kα X-ray source at 225 Watts. For examining the morphology and structural features, a JEOL JSM-7900F field-emission scanning electron microscopy (FESEM) instrument was employed. To assess the textural properties of the sample, BET analysis was performed *via* nitrogen adsorption–desorption experiments at 77 K using an Anton Paar Autosorb 6100 FKM MP-AG instrument. It provided detailed information about the total specific surface area and pore size accessible within the sample. The CorrTest (CS2350M) electrochemical workstation was employed to conduct a systematic examination of the electrochemical characteristics of the synthesized electrodes.

### 2.7 Electrochemical analysis

The experimental setup utilized a three-electrode configuration, with Co-MOF on a nickel substrate as the working electrode, Ag/AgCl electrode as the reference electrode, and a platinum wire as the counter electrode, and 1 M KOH and 1 M NaOH aqueous electrolyte solution. Electrochemical analyses, including CV, GCD and EIS, were conducted. For the CV measurements, a voltage window of 0 V to 0.8 V was used and the materials were tested at scan rates up to 100 mV s^−1^. The GCD experiments were conducted over a range of *I*_d_, up to 10 A g^−1^, to evaluate performance under different conditions. The EIS measurements were performed by sweeping the frequency from 100 kHz down to 10 mHz. Therefore, the values of *C*_sp_, *E*_d_ and *P*_d_ were evaluated based on the following electrochemical relations:^69^1
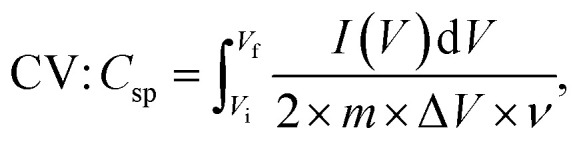
2
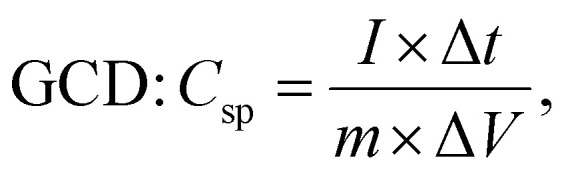
3
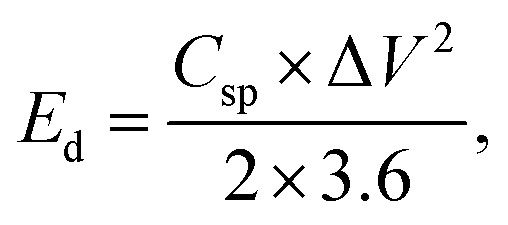
4
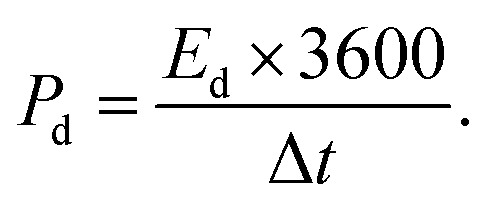
In these expressions, *C*_sp_ (F g^−1^) denotes the specific capacitance and Δ*V* corresponds to the voltage range. The term 
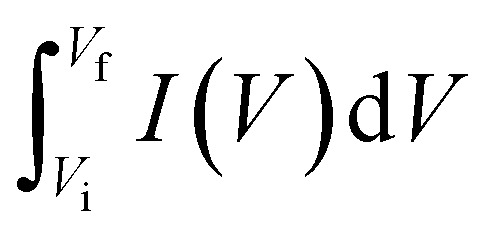
 denotes the area integrated from the CV profile, while *I* indicates the applied current, and *m* represents the total weight of the active material. Additionally, *ν* corresponds to the scan rate, Δ*t* represents discharge duration, and *P*_d_ and *E*_d_ denote the power and energy density, respectively.^70^

The electrochemical parameters of the symmetric supercapacitor, including *C*_cell_, *E*_cell_ (W h kg^−1^) and *P*_cell_ (W kg^−1^), were calculated using the following formulas:^71,72^5
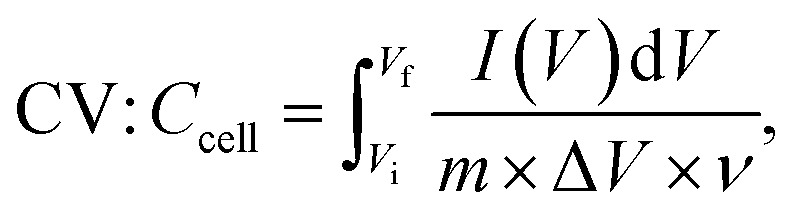
6
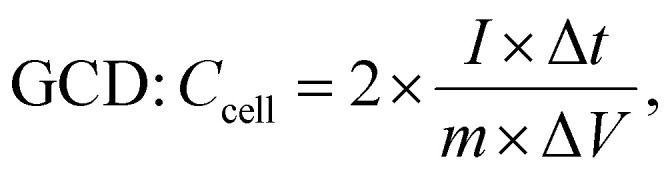
7
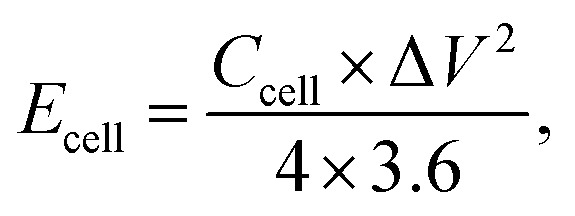
8
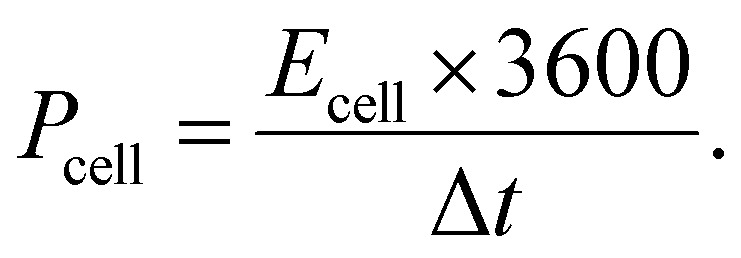


## 3. Results and discussion

### 3.1 Characterization of the powder sample

**Fig. 2:**
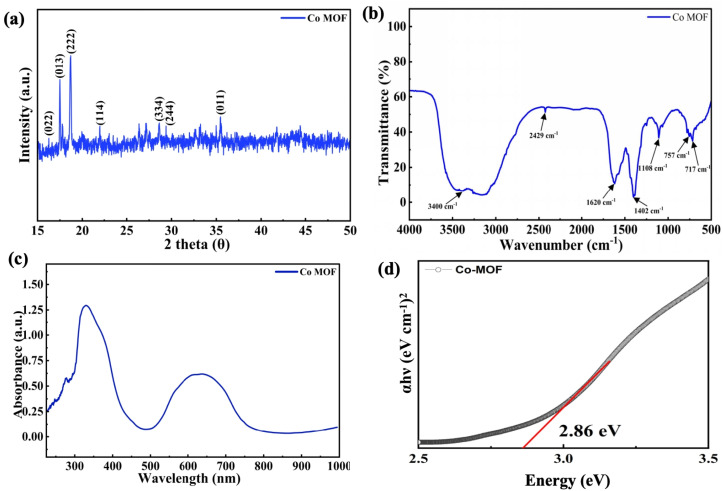
(a) PXRD pattern of Co-MOF. (b) FTIR spectrum of Co-MOF. (c) UV-vis spectrum of Co-MOF. (d) *E*_g_ plot of Co-MOF.

The crystallinity and phase purity of the Co-MOF were evaluated through PXRD analysis, as presented in Fig. 2(a), demonstrating that the structure was highly crystalline. The peaks for Co-MOF appeared at 16.27°, 17.49°, 18.70°, 21.95°, 28.63°, 29.39°, and 35.4°, corresponding to planes (022), (013), (222), (114), (334), (244) and (011), respectively.^73^ The XRD patterns of Co-MOF showed two main peaks at 17.49° and 18.70°, which correspond to a standard reference pattern, confirming that the observed diffraction peaks of the synthesized Co-MOF matched the previously reported MOF (CCDC no. 245714).^73^ These results show that the synthesized Co-MOF was successfully prepared without any impurity.

To examine the functional groups within the Co-MOF, the material was analyzed *via* FT-IR analysis and is shown in Fig. 2(b). The absorption band at 3400 cm^−1^ corresponds to the O–H stretching, suggesting the presence of hydroxide functional groups associated with the ligands and linker molecules within the Co-MOF framework. Moreover, the peak at 2429 cm^−1^ indicates the hydroxyl functional groups, which can be due to absorbed moisture bonded to the MOF surface. Peaks at 1620 cm^−1^ and 1402 cm^−1^ were attributed to the asymmetric and symmetric vibrations of the carboxylate functional group. The observed band at 1055.81 cm^−1^ shows the C–O stretching vibration, which means that carbonyl groups (C=O) are present. The absorption peaks observed at 618–771 cm^−1^ were assigned to C–H bending in the MOF structure. The prominent absorption peak observed around 717 cm^−1^ was attributed to Co–O stretching vibrations, confirming coordination between the cobalt ions and the organic linker within the framework.

The optical characteristics of the synthesized Co-MOF were assessed through ultraviolet-visible diffuse reflectance spectroscopy (UV-DRS) analysis. The absorption spectrum in Fig. 2(c) shows two prominent peaks at ∼328.9 nm and 627.4 nm, corresponding to ligand-to-metal charge transfer (LMCT) and d–d transitions of Co(ii) centers, respectively, in alignment with the previous literature. A small absorption peak around 280 nm can be associated with the π–π* electronic transition. Similarly, the band gap value can be determined using the Tauc equation as follows:

9 (*αhν*)^*n*^ = *β*(*hν* − *E*_g_).

Here, *α* represents the absorption coefficient, *β* denotes a constant in the Tauc relation, *hν* signifies the energy of the photon involved in the absorption process, *E*_g_ indicates the band gap energy, where *n* corresponds to 2 and 0.5 for direct and indirect allowed transitions, respectively. The direct *E*_g_ of the Co-MOF was evaluated to be 2.86 eV, as displayed in Fig. 2(d)*via* the plot of (*αhν*)^*n*^*versus hν*, indicating the potential for optical applications.

**Fig. 3:**
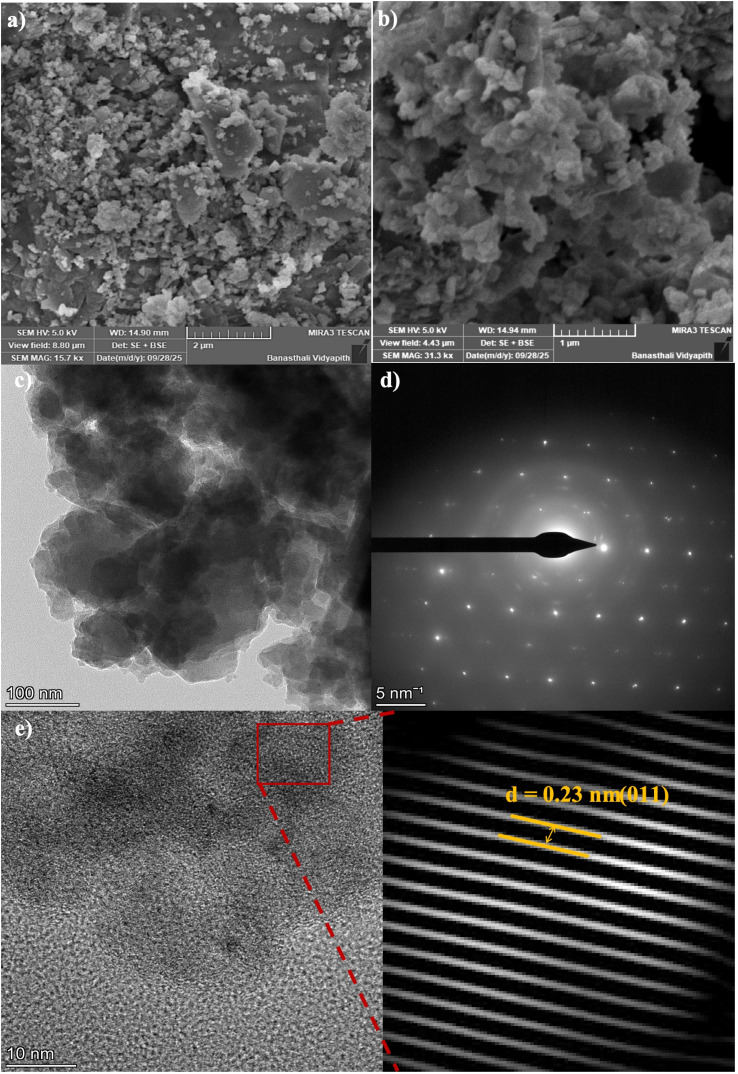
(a and b) FE-SEM images of Co-MOF. (c) HR-TEM image of Co-MOF. (d) SAED pattern of Co-MOF. (e) FFT patterns of Co-MOF.

The FE-SEM images in Fig. 3(a) and (b) show that the as-synthesized Co-MOF consists of irregularly shaped micro-aggregates with a rough morphology. The micrographs displayed densely packed granular formations made of interconnected nanoscale crystallites. In various regions, flake and plate-like structures were observed, signifying non-uniform crystal development. This morphology indicates heterogeneous nucleation and growth during synthesis, resulting in a porous and loosely packed structure that could enhance electrolyte access and electrochemical activity.

Fig. 3(c) presents the HR-TEM micrograph of the Co-MOF, while Fig. 3(d) displays the SAED pattern of the Co-MOF, where distinct diffraction rings were observed. Fig. 3(e) shows a magnified view of the same image, which was used to determine the interplanar spacing *d*. The HR-TEM analysis revealed clear lattice fringes, indicating that the (011) plane of the Co-MOF exhibited a *d*-spacing of approximately 0.23 nm. These findings confirmed the crystalline nature and structural framework of the Co-MOF.^74–77^

**Fig. 4:**
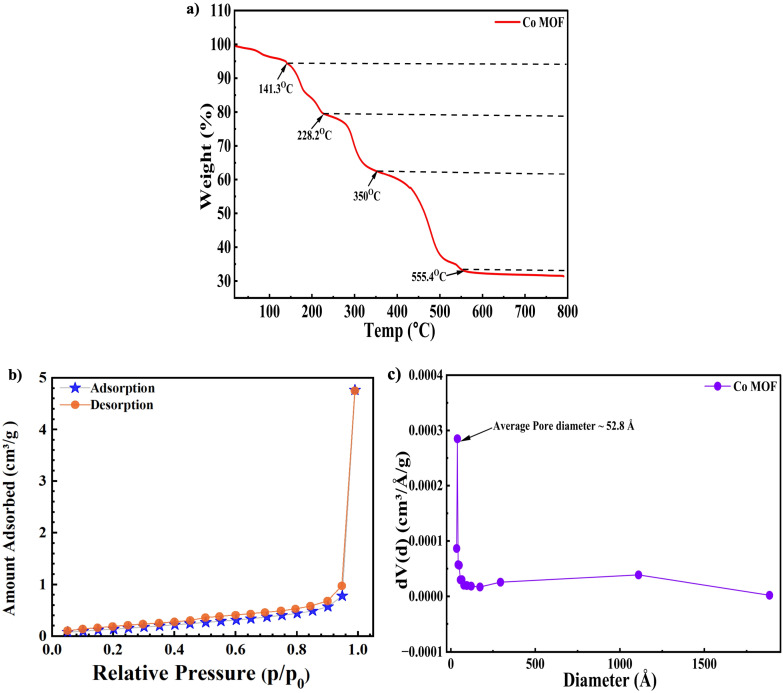
(a) TGA analysis of Co-MOF. (b) BET isotherm analysis of Co-MOF. (c) Pore size distribution analysis of Co-MOF.

To further analyze the decomposition characteristics and thermal stability of the Co-MOF precursor, thermogravimetric analysis (TGA) was conducted with the data presented in Fig. 4(a). The thermal conversion of Co-MOF occurs through several stages of weight loss. An initial mass loss of approximately 5% below 141.3 °C was attributed to the removal of physically adsorbed moisture, with any remaining DMF solvent trapped within the structure. A further 15% weight loss around 228.2 °C was observed, associated with the loss of lattice-bound water molecules. An additional decrease of approximately 18% took place between 228.2 °C and 350 °C, attributed to the gradual evaporation and decomposition of coordinated BTC. The most substantial weight loss, about 29%, observed in the range of 350 °C and 555.4 °C, corresponds to the collapse of the Co-MOF structure and its transformation into cobalt oxide. Beyond 555.4 °C, negligible mass change was observed, signifying the completion of the MOF to oxide transformation.^78^

Nitrogen adsorption–desorption analyses were also conducted to assess the porosity and surface area of the Co-MOF sample. As illustrated in Fig. 4(b), the resulting isotherm exhibited a characteristic type-4 profile accompanied by an H4-type hysteresis pattern, indicating the presence of mesopores.^78^ BET analysis revealed that the sample displayed a specific surface area measuring 5.452 m^2^ g^−1^, while the average pore diameter was found to be 52.8 Å, as shown in Fig. 4(c); a higher pore volume significantly improves the electrochemical performance by providing a larger surface area for interaction with the electrolyte.^79–81^ This also creates more accessible sites for ion intercalation, enabling smoother charge transport and ultimately enhancing the overall electrochemical behaviour. However, maintaining a moderate surface area can reduce unwanted reactions with the electrolyte, thus enhancing the stable electrochemical performance.^82^

**Fig. 5:**
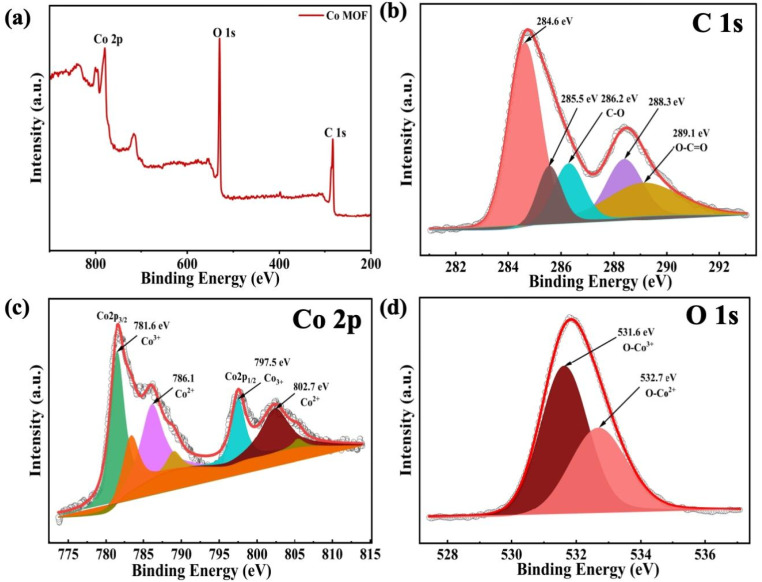
XPS analysis of Co-MOF: (a) survey scan spectrum and high-resolution (b) C 1s spectrum, (c) Co 2p spectrum, and (d) O 1s spectrum.

XPS analysis was subsequently employed to evaluate the elemental composition and respective oxidation states present in the Co-MOF. The survey spectrum depicted in Fig. 5(a) clearly indicates that Co-MOF predominantly consists of Co, O, and C elements. The C 1s spectrum shown in Fig. 5(b) displays three peaks at binding energies (B.E.) of 284.6, 286.2, and 289.1 eV, which correspond to C–C, C–O, and, C=O, functional groups, respectively. Furthermore, the peaks observed at 285.5 eV and 288.3 eV were associated with satellite features. The high-resolution Co 2p spectrum was characterized by two dominant peaks located at B.E. of 781.6 eV and 797.5 eV, which were attributed to the Co 2p_3/2_ and Co 2p_1/2_ spin–spin orbitals, respectively. The observed spin–orbit separation of 15.9 eV was consistent with the electronic structure of cobalt species as shown in Fig. 5(c). In addition, the separation of peaks indicates that Co predominantly exists in the +2 oxidation state within the Co-MOF. The slightly elevated B.E. compared to previously reported values is attributed to strong coordination interactions between Co^2+^ ions and carboxylate (–COO^−^) ligands in the MOF structure.^82^ The O 1s exhibited two distinct peaks, as shown in Fig. 5(d), indicating that oxygen exists in different chemical environments. The observed peak centered at the B.E. of 531.6 eV is associated with the carboxylate (O–Co) functional group, while the peak located at 532.7 eV confirms metal–ligand bonding, *i.e.*, the coordination between oxygen and cobalt atoms.

### 3.2 Electrochemical evaluation of Co-MOF using a three-electrode setup

**Fig. 6:**
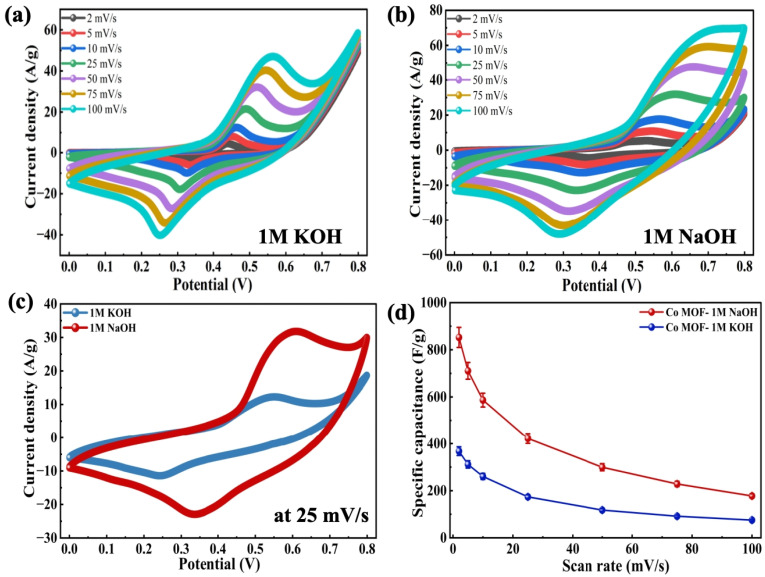
CV curves of Co-MOF in (a) 1 M KOH and (b) 1 M NaOH. (c) Comparison graphs of the CV analysis of Co-MOF in 1 M KOH and 1 M NaOH at 25 mV s^−1^. (d) *C*_sp_*vs.* scan rate plot for Co-MOF in 1 M KOH and 1 M NaOH.

The Co-MOF was systematically investigated in 1 M KOH and 1 M NaOH electrolytes to evaluate its electrochemical behaviour. The CV profiles of Co-MOF electrodes fabricated in these respective electrolytes are presented in Fig. 6(a) and (b). The measurements were performed in the voltage range between 0 and 0.8 V and sweep rates between 5 to 100 mV s^−1^. The Co-MOF electrodes were typically performed over a wider potential range to display redox peaks due to Co centers in the MOF undergoing reversible oxidation–reduction reactions associated with the reversible faradaic transition and pseudocapacitive behaviour. Even at higher scan rates, the CV profiles exhibited minimal distortion and retained their characteristic shape, demonstrating excellent capacitive performance resulting from rapid ion diffusion within the composite.^83^ The effect of electrolyte cations on the electrochemical performance of the active material was further investigated using two separate aqueous electrolytes, KOH and NaOH. Fig. 6(c) compares the CV responses of the electrodes in two different electrolytes at a potential sweep rate of 25 mV s^−1^. The Co-MOF electrode showed the highest *C*_sp_ of 852.5 F g^−1^ at a potential sweep rate of 2 mV s^−1^ in 1 M NaOH, while in 1 M KOH, it reached 379.31 F g^−1^ at the same scan rate. The CV curve of the Co-MOF electrode measured in 1 M NaOH and 1 M KOH exhibited distinct variations, with a distinct pattern, along with a significantly larger CV area observed in NaOH relative to KOH. These results signify enhanced charge storage performance in the NaOH electrolyte when compared with KOH, as presented in Fig. 6(d). A comprehensive overview of *C*_sp_ as a function of scan rate is tabulated in Table 1. The comparative CV analysis revealed that NaOH was the most suitable electrolyte, delivering maximum efficiency. This behaviour can be attributed to the effects of electrolyte cations on electrochemical performance, observed by examining the hydrated ionic radii and ionic dimensions as essential parameters. In this context, the comparison of hydrated ionic radii showed that K^+^ ions (∼0.3 nm) are smaller than Na^+^ ions (∼0.4 nm), which influences their mobility and interaction with the electrode. The variations in hydrated ionic radii directly influenced ion diffusion kinetics and consequently, the overall charge storage characteristics of the active material. Therefore, KOH exhibited a lower *C*_sp_ than NaOH. This difference can be further explained by examining the intrinsic ionic radii of the electrolyte cations, which also significantly influence charge transport. The K^+^ ion exhibits a larger ionic radius of 0.138 nm compared to the Na^+^ ion, which has a radius of 0.102 nm, resulting in K^+^ ions diffusing less effectively towards the electrode. Consequently, the synergistic impact of the hydrated ionic radii and ionic dimensions resulted in the enhanced efficacy of NaOH relative to KOH.

**Table 1:** Comparison of the *C*_sp_ values at different scan rates for the electrodes tested in aqueous 1 M NaOH and 1 M KOH solutions

Electrolyte	Specific capacitance (F g^−1^) values at different scan rates (mV s^−1^)						
	2	5	10	25	50	75	100
1 M NaOH	852.5	710.75	586.5	422.32	299.62	228.59	181.9
1 M KOH	379.31	295.49	255.54	209.01	165.19	139.26	123.36

**Fig. 7:**
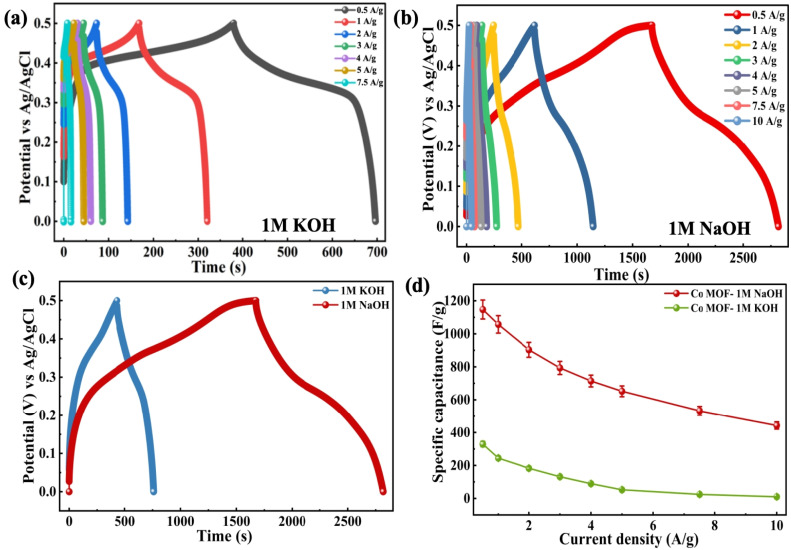
GCD curves of Co-MOF in (a) 1 M KOH and (b) 1 M NaOH. (c) Comparison graphs of the GCD analysis of Co-MOF in 1 M KOH and 1 M NaOH at 0.5 A g^−1^. (d) *C*_sp_*vs. I*_d_ graphs of Co-MOF in 1 M KOH and 1 M NaOH.

**Fig. 8:**
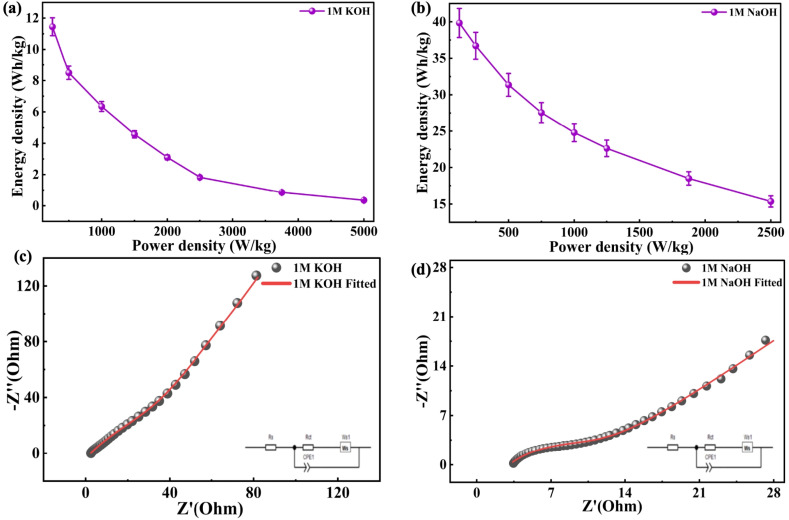
Ragone plots of Co-MOF in (a) 1 M KOH and (b) 1 M NaOH. EIS graphs of Co-MOF in (c) 1 M KOH and (d) 1 M NaOH.

Furthermore, the electrode *C*_sp_ properties were assessed by employing the GCD method. The charge–discharge profiles presented in Fig. 7(a) and (b) display non-linear variations, which reflect the pseudocapacitive characteristics of the electrode material, aligning with the GCD results for both 1 M KOH and 1 M NaOH. The data were obtained over the voltage range of 0–0.5 V, employing varying *I*_d_, ranging from 0.5 to 10 A g^−1^ in both electrolytes. The GCD measurements were carried out within a narrower potential window to avoid undesirable side reactions, such as electrolyte decomposition or structural degradation of the electrode, which occur at higher potentials under constant current conditions. Additionally, in extended voltage ranges, *iR* drop and polarization effects appear more clearly in GCD, which distort the charge–discharge profiles and affect the accuracy of capacitance calculations. Fig. 7(c) compares the discharge times at 0.5 A g^−1^, demonstrating that maximum discharge duration occurs with 1 M NaOH, signifying higher *C*_sp_. The *C*_sp_ determined from the GCD measurements using eqn (11) is plotted against *I*_d_ in Fig. 7(d). The Co-MOF electrode achieved a peak *C*_sp_ of 1147.2 F g^−1^ when tested at 0.5 A g^−1^ in 1 M NaOH, highlighting its superior electrochemical behaviour in NaOH relative to KOH electrolyte. Among the electrolytes investigated, the Co-MOF electrode operating in 1 M NaOH demonstrated the highest performance, achieving an *E*_d_ of 39.83 W h kg^−1^, while the *P*_d_ of 125 W kg^−1^ was obtained using eqn (3) and (4). The values considerably exceed those observed for KOH, which delivered an *E*_d_ of 11.04 W h kg^−1^ and *P*_d_ of 125 W kg^−1^, as shown in Fig. 8(a) and (b).

Following the GCD analysis, EIS was conducted to analyze the charge mobility characteristics and interfacial kinetics of the Co-MOF electrodes. EIS is particularly valuable for understanding resistance contributions and ion diffusion behaviour, which are essential factors influencing the power performance of supercapacitor electrodes. The intersection of the semi-circular loop with the *x*-axis in a Nyquist plot demonstrates the ohmic resistance at the electrode and the electrolyte junction.^84^

Consequently, Nyquist plots were obtained for Co-MOF electrodes tested in both KOH and NaOH electrolytes and examined from 100 kHz to 0.01 Hz, as shown in Fig. 8(c) and (d). Two characteristic regions were observed, consisting of a semi-circular loop in the high-frequency range and a sloped line appearing at low frequencies. The first corresponds to interfacial charge transfer processes occurring during the redox reaction between the electrolyte/electrode interface; the second indicates the mobility of ions and capacitive charge storage within the electrode. The point where the high-frequency intersects the real axis corresponds to the solution resistance (*R*_s_), while the width of the semicircle indicates the charge-transfer resistance (*R*_ct_). The Co-MOF electrode demonstrated *R*_s_ of 3.05 Ω in 1 M NaOH, which was slightly higher than the 2.28 Ω observed in 1 M KOH. However, *R*_ct_ in 1 M NaOH (10.41 Ω) was significantly lower than that measured in 1 M KOH (158.08 Ω). This suggests that 1 M NaOH electrolyte facilitates a more efficient *R*_ct_ at the electrode–electrolyte interface, despite a minor difference in *R*_s_. Furthermore, the more prominent inclined linear response observed in the low-frequency region signifies improved capacitive performance resulting from effective ion accumulation and diffusion. Overall, the lower solution and charge transfer resistances in NaOH accounted for its superior electrochemical and capacitive performance compared to KOH.

### 3.3 Electrochemical behaviour of Co-MOF in three-electrode setups for varying molarities

**Fig. 9:**
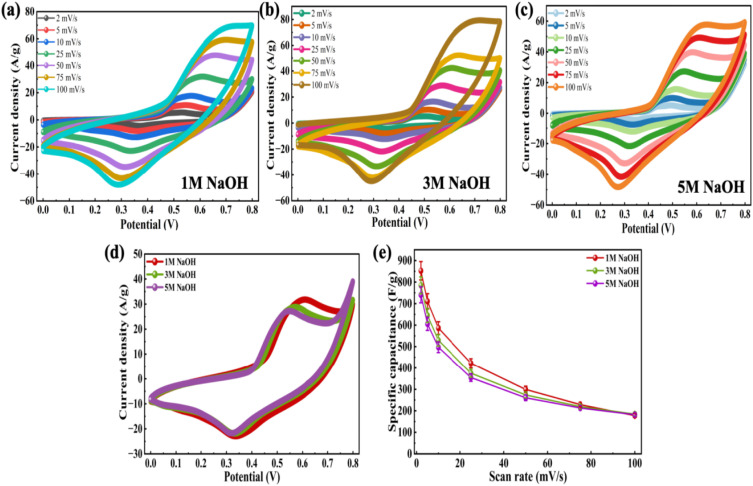
CV profiles of Co-MOF in (a) 1 M NaOH, (b) 3 M NaOH, and (c) 5 M NaOH. (d) Comparison graphs of Co-MOF in 1 M NaOH, 3 M NaOH, and 5 M NaOH at 25 mV s^−1^. (e) *C*_sp_*vs.* scan rate plots of Co-MOF in 1 M NaOH, 3 M NaOH and 5 M NaOH.

Since the Co-MOF demonstrated the best performance with NaOH as the electrolyte, its concentration was subsequently varied to 1 M, 3 M and 5 M, as shown in Fig. 9(a)–(c). A comparative analysis of all the electrolyte concentrations was carried out using CV, where the responses obtained at a potential sweep rate of 25 mV s^−1^ are presented in Fig. 9(d). The Co-MOF electrode evaluated in 1 M NaOH exhibited a significantly higher peak current compared to those measured in electrolytes of different molarities (3 M and 5 M NaOH). This improved current response was demonstrated by the larger area enclosed within the CV curves, which corresponds to increased *C*_sp_ values. The Co-MOF electrode exhibited *C*_sp_ of 852.5 F g^−1^, 784.68 F g^−1^ and 740.87 F g^−1^ in 1 M, 3 M and 5 M NaOH, respectively, as shown in Fig. 9(e). Additionally, Dunn's method was used to provide a comprehensive analysis to understand the mechanisms of charge storage in the Co-MOF with various electrolytes. Alkaline KOH and NaOH electrolytes were analyzed to differentiate between charge storage arising from ion diffusion within the bulk material occurring at the electrode surface. The *b*-values were derived from CV data by examining the variation of current (*I*) with scan rate (*ν*), utilizing eqn (10) and (11) as follows:^85^

10 *I* = *I*_c_ + *I*_d_ = *aν*^*b*^,

11 log *I* = *b* log *ν* + log *a*.

**Fig. 10:**
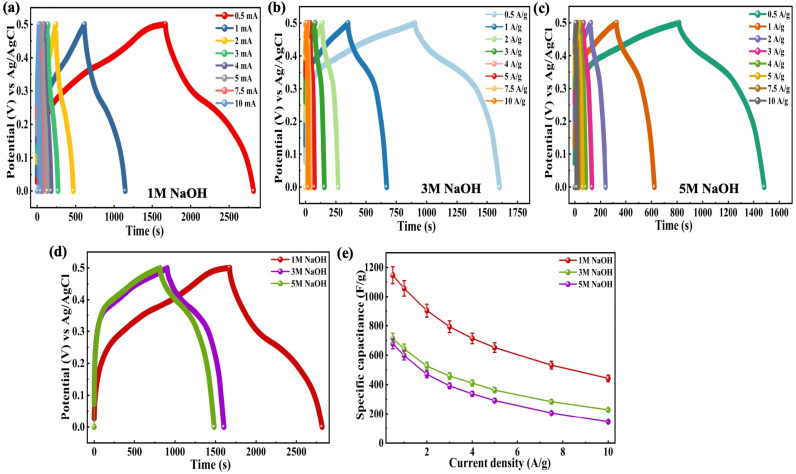
GCD curves of Co-MOF in (a) 1 M NaOH, (b) 3 M NaOH, and (c) 5 M NaOH. (d) Comparison graph of Co-MOF in 1 M NaOH, 3 M NaOH and 5 M NaOH at 0.5 A g^−1^. (e) *C*_sp_*vs. I*_d_ plots of Co-MOF in 1 M NaOH, 3 M NaOH and 5 M NaOH.

Here, *I* denotes the total current response, and *I*_c_ and *I*_d_ correspond to the current arising from surface-controlled and diffusion-controlled mechanisms. The slope of the line was obtained by constructing a plot of log(*I*) and log(*ν*), which corresponds to the value of *b* and provides insight into whether the charge storage in the electrode is controlled predominantly by ion diffusion. In this context, a value of *b* approaching 1 suggests capacitive behaviour, while a value close to 0.5 suggests diffusion-controlled behaviour.^85^ Intermediate *b*-values imply the simultaneous occurrence of both processes; the respective log(*I*)–log(*ν*) plots for the Co-MOF electrodes in KOH, NaOH and their various molarities are shown in Fig. S2(a). At a concentration of 1 M KOH and NaOH, the calculated *b*-values were 0.61 and 0.63, respectively; for 3 M and 5 M NaOH, they were 0.65 and 0.61, respectively. Since all these values lie between 0.5 and 1, this indicates that the charge storage mechanism is governed by synergistic contributions from diffusion-controlled faradaic reactions and surface-controlled capacitive processes. To gain deeper insight into the charge storage behaviour at the electrode–electrolyte interface, the current values at peak potentials were extracted from the CV curves recorded at different scan rates. This approach shows the individual contributions to the overall charge storage. The relative contributions of surface and diffusion processes to the total capacitance vary with scan rate. With an increase in scan rate, the diffusion-controlled contribution gradually decreased, while the surface-controlled redox processes became more dominant for Co-MOF in KOH and NaOH and their varied molarity, as illustrated in Fig. S2(b)–(e). This behaviour suggests that ion transport was more effective at the electrode surface at higher scan rates, highlighting that charge storage occurred due to both mechanisms, which ultimately influences the overall electrochemical performance of the electrode.^55^ Subsequently, GCD measured under different molarities were analyzed for various NaOH molarities as shown in Fig. 10(a)–(c). Among these, the 1 M NaOH electrolyte demonstrated the longest discharge duration, indicating superior charge storage performance relative to the electrolytes with higher molarities. This improved performance can be due to enhanced ion mobility and effective use of electroactive sites at an optimal electrolyte concentration, thereby enabling faster and more reversible redox reactions as illustrated in Fig. 10(d), comparison graph between different molarities at 0.5 A g^−1^*I*_d_. This trend was further validated by GCD analysis conducted across a range of applied *I*_d_'s as displayed in Fig. 10(e), where the corresponding *C*_sp_ of 1147 F g^−1^, 714 F g^−1^ and 677 F g^−1^ were observed for 1 M, 3 M, and 5 M NaOH, respectively.

**Fig. 11:**
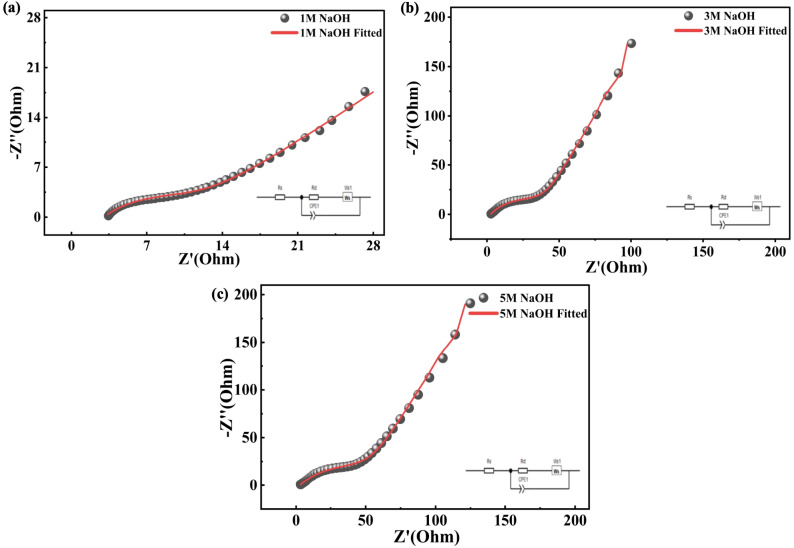
Fitted EIS graphs of Co-MOF in (a) 1 M NaOH, (b) 3 M NaOH, and (c) 5 M NaOH.

Subsequently, the charge storage processes and the kinetics of the interfacial charge transfer characteristics of the Co-MOF electrodes were studied using EIS. Nyquist plots were obtained in 1 M, 3 M and 5 M NaOH as depicted in Fig. 11(a)–(c). Among the electrolytes examined, the Co-MOF electrode in 1 M NaOH demonstrated the smallest semi-circular arc at high frequencies, signifying the lowest overall impedance and more effective movement of charge across the interface of the electrolyte and electrode. Quantitative analysis of the impedance spectra indicated that the electrode in 1 M NaOH exhibited a *R*_s_ of 3.05 Ω and *R*_ct_ of 10.41 Ω, both of which were significantly lower than those observed in 3 M NaOH (*R*_s_ = 2.54 Ω and *R*_ct_ = 48.90 Ω) and 5 M NaOH (*R*_s_ = 2.85 Ω and *R*_ct_ = 70.72 Ω). The significantly decreased *R*_ct_ in 1 M NaOH could be due to enhanced ionic mobility and efficient electrolyte penetration within the porous Co-MOF framework, promoting rapid electron and ion transport during electrochemical processes. Conversely, higher electrolyte concentrations tend to promote enhanced ion–ion interactions and viscosity effects, which hinder ion diffusion and decrease interfacial charge transfer. Therefore, the improved impedance characteristics observed in 1 M NaOH promoted enhanced electrochemical performance and faster charge transfer kinetics for the Co-MOF electrode. Moreover, the cycling stability of the Co-MOF electrode demonstrated exceptional durability in 1 M NaOH, with a coulombic efficiency of approximately 98% after 10 000 cycles. Table 2 presents a comparative analysis of CV, GCD and EIS characteristics obtained using NaOH electrolytes of varying molarities (1 M, 3 M and 5 M).

**Table 2:** Effects of the molarity of the NaOH electrolyte (1 M, 3 M and 5 M) on CV, GCD, and EIS analyses

Electrolyte molarity (NaOH)	CV (at 2 mV s^−1^)	GCD (at 0.5 A g^−1^)	Resistance	
			*R*_s_	*R*_ct_
1 M	852.5 F g^−1^	1147 F g^−1^	3.05 Ω	10.41 Ω
3 M	784.68 F g^−1^	714 F g^−1^	2.54 Ω	48.90 Ω
5 M	740.87 F g^−1^	677 F g^−1^	2.85 Ω	70.72 Ω

Table 3 compares the electrochemical performance of this study with previously published literature, highlighting the current findings.

**Table 3:** Reported electrochemical performances of Co-MOF-based electrodes with different synthesis routes and electrolyte molarities

S. no.	Material	Electrolyte molarity	Synthesis	Specific capacitance	Cyclic stability	Ref.
1	Co-based MOF	1 M KOH	Solvothermal	980 F g^−1^	72.38% after 2000 cycles	88
2	ZIF-67 MOF	3 M KOH	Electrodeposition	105 mF cm^−2^	>80% after 10 000 cycles	89
3	ZIF-67	6 M KOH	—	1068.62 F g^−1^	84.98% after 6000 cycles	90
4	CTNBMs	1 M NaOH	Hydrothermal	472.8 F g^−1^	85.9% after 6000 cycles	91
5	Co-MOF	6 M KOH	—	316 F g^−1^	—	92
6	Co-MOF	1 M KOH	Solvothermal	571.73 F g^−1^	96.87% after 5000 cycles	52
7	Co-MOF	2 M KOH	Hydrothermal	261.27 F g^−1^	84% after 2000 cycles	93
**8**	Co-MOF	**1 M NaOH**	**Hydrothermal**	**1147 F g^−^** ^**1**^	**98% after 10 000 cycles**	**This work**

### 3.4 Electrochemical evaluation of the Co-MOF symmetric supercapacitor

**Fig. 12:**
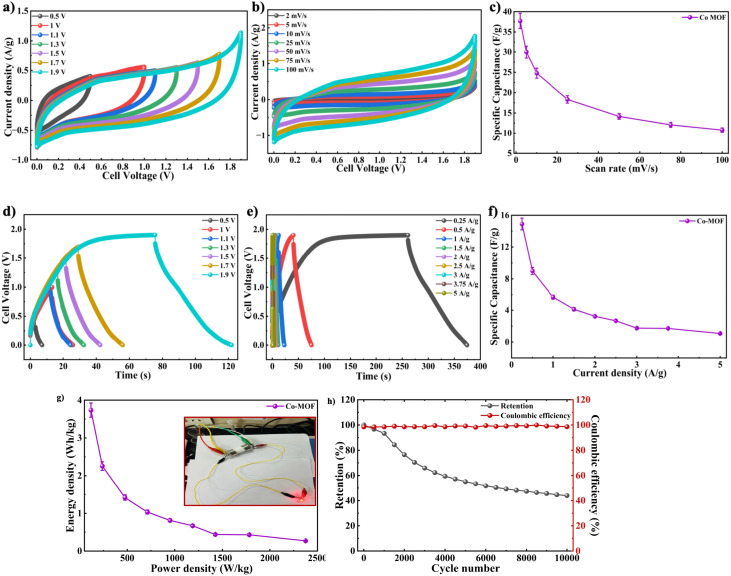
(a) Operating voltage optimization analysis using CV. (b) CV profiles of the Co-MOF Swagelok device. (c) *C*_sp_*vs.* scan rate graph of Co-MOF. (d) Operating voltage optimization analysis using GCD. (e) GCD plots of the Co-MOF Swagelok device. (f) Variation of *C*_sp_ with *I*_d_. (g) Ragone analysis of the Co-MOF Swagelok device; inset: LED illumination by the Co-MOF Swagelok device. (h) Cyclic stability analysis of the Co-MOF Swagelok device.

After confirming the electrochemical performance of the Co-MOF electrode in 1 M NaOH electrolyte using a three-electrode configuration, its practical applicability was further evaluated by assembling a symmetric supercapacitor device in a Swagelok-type cell. The supercapacitor device consisted of a pair of identical Co-MOF electrodes with a separator positioned between them. The fabricated device was systematically evaluated for its electrochemical characteristics in 1 M NaOH through CV and GCD analysis. The operating voltage window of the Co-MOF Swagelok cell was determined to be 1.9 V from CV measurements in different potential windows with a sweep rate of 100 mV s^−1^, as displayed in Fig. 12(a). The CV responses of the Co-MOF-based symmetric supercapacitor device measured at scan rates varied from 2 to 100 mV s^−1^ showed a quasi-rectangular profile with noticeable curves at higher potentials. It broadened gradually with increasing scan rates, accompanied by a systematic rise in *I*_d_, indicating enhanced charge storage capability and efficient electrochemical behaviour. Although the curves did not display prominent redox peaks, the deviation from an ideal rectangular shape indicated the occurrence of surface-controlled faradaic reactions alongside the electric double-layer capacitance, as shown in Fig. 12(b). The *C*_sp_ values were calculated from the graph in Fig. 12(c), which displays the *C*_sp_ of 37.7 F g^−1^ achieved at a scan rate of 2 mV s^−1^. As the scan rate increased, the *C*_sp_ gradually decreased to 30 F g^−1^ at 5 mV s^−1^ and subsequently to 24.78 F g^−1^, 18.33 F g^−1^, 14.15 F g^−1^, 12.07 F g^−1^ and 10.78 F g^−1^ at scan rates of 10 mV s^−1^, 25 mV s^−1^, 50 mV s^−1^, 75 mV s^−1^ and 100 mV s^−1^, respectively. This decline in capacitance, observed at increased scan rates, could be caused by kinetic limitations, whereby electrolyte ions were unable to thoroughly penetrate the internal porous structure of the Co-MOF, thereby limiting charge storage mainly to surface-accessible active sites. The consistent CV profiles observed across all scan rates indicated the good rate capability and electrochemical stability of the Co-MOF electrode in the symmetric Swagelok cell. To determine the optimal potential window, the GCD of the Co-MOF Swagelok cell was performed in the voltage window range of 0.0–1.9 V, as shown in Fig. 12(d). The GCD profiles obtained at different *I*_d_'s within the range of 0.25–5 A g^−1^ over the same operating potential window as the CV measurements are shown in Fig. 12(e). The quasi-symmetric triangular profile confirmed the effective charge storage and highly reversible electrochemical behaviour of the device. The *C*_sp_ values calculated using the eqn (7) reached a peak *C*_sp_ of 14.9 F g^−1^ measured at an *I*_d_ of 0.25 A g^−1^, as depicted in Fig. 12(f). The comparatively low coulombic efficiency noted at an *I*_d_ 0.25 A g^−1^ was due to side reactions occurring at lower *I*_d_. The extended charge–discharge period at low *I*_d_ facilitated electrolyte decomposition and potential gas evolution in the aqueous medium. Furthermore, the prolonged ion diffusion duration may have caused partial irreversibility in faradaic reactions and charge trapping within the electrode structure. These factors collectively resulted in reduced charge recovery and lower coulombic efficiency. With increasing *I*_d_, a noticeable decrease in capacitance was observed. Specifically, the capacitance values were measured at 0.5 A g^−1^, 1 A g^−1^, 1.5 A g^−1^, 2 A g^−1^, 2.5 A g^−1^, 3 A g^−1^ and 3.75 A g^−1^ and 5 A g^−1^, showing a progressive decline as the *I*_d_ increased. This reduction in capacitive behaviour at increased *I*_d_s is likely due to increased internal resistance and limited duration for ions to penetrate the electroactive sites, thereby limiting the charge storage efficiency.^86^

This was further evaluated by calculating *E*_d_ and *P*_d_ using Ragone analysis, as shown in Fig. 12(g). A maximum *P*_d_ peak of 118.75 W kg^−1^ was achieved along with an *E*_d_ of 3.73 W h kg^−1^ by the Swagelok cell. To demonstrate practical applicability, the assembled Swagelok supercapacitor device was effectively employed to power a red LED, which remained illuminated for approximately 2 minutes as illustrated in inset image of Fig. 12(g), highlighting the potential of the Co-MOF electrode for practical energy storage devices. The cycling stability of the Co-MOF electrode was assessed and subjected to 10 000 continuous cycles at 1 A g^−1^ with the corresponding coulombic efficiency of 98.66% and retention of 43.96%, as illustrated in Fig. 12(h). A gradual decrease in capacitance over prolonged cycling can be attributed to factors such as the partial irreversibility of redox reactions, electrolyte degradation, loss of active material and thermal effects, which collectively restrict ion transport and reduce the electrochemically active surface area.^87^ Nevertheless, the device demonstrated consistent cycling performance.

**Fig. 13:**
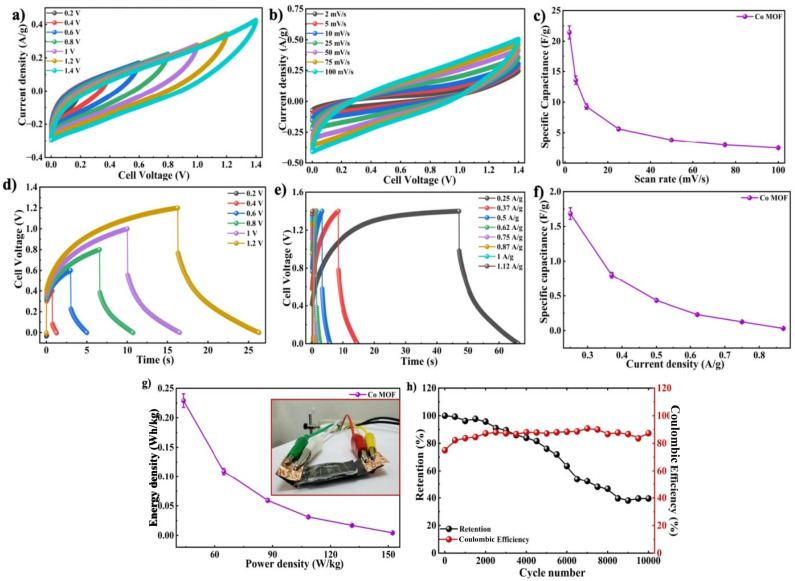
(a) Operating voltage optimization analysis using CV. (b) CV curves of the Co-MOF pouch cell device. (c) *C*_sp_*vs.* scan rate graph. (d) Operating voltage optimization analysis using GCD. (e) GCD plots of the Co-MOF pouch cell device. (f) Variation of *C*_sp_ with *I*_d_. (g) Ragone plot of the Co-MOF pouch cell; inset: Co-MOF pouch cell. (h) Cyclic stability analysis of the Co-MOF pouch cell.

Similarly, the electrochemical performance of the Co-MOF pouch cell was evaluated using CV, GCD, and cycling stability tests in a two-electrode setup. The operating voltage limits of the device were examined by performing CV at 50 mV s^−1^, with a gradually applied potential from 0 V to 1.4 V, as shown in Fig. 13(a). Therefore, voltage limits of 0.2 V, 0.4 V, 0.8 V, 1 V, 1.2 V, and 1.4 V were selected as the working potential windows for all further measurements. As shown in Fig. 13(b), the CV measurements were carried out over the specified optimal potential window at scan rates between 2 and 100 mV s^−1^. At 2 mV s^−1^, the Co-MOF pouch cell delivered a substantial *C*_sp_ of 21.42 F g^−1^, as depicted in Fig. 13(c). Rapid charge propagation and characteristic capacitive behaviour of the Co-MOF-based device were confirmed by the steady enhancement in current response observed at higher scan rates.

**Fig. 14:**
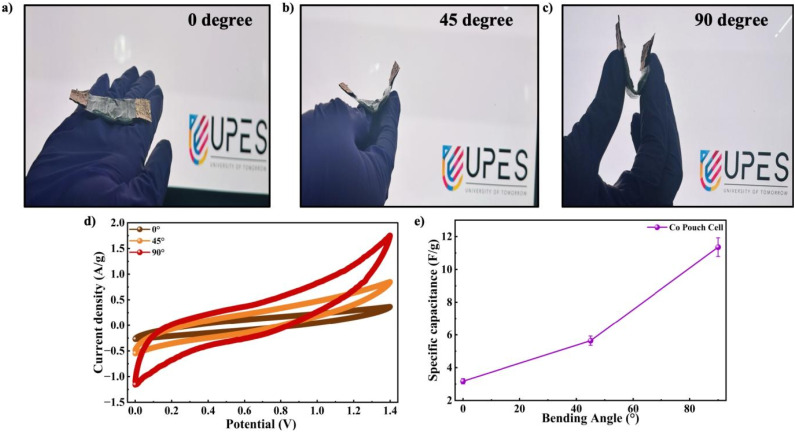
Bending tests of the Co-MOF pouch cell at (a) 0°, (b) 45°, and (c) 90°. (d) Comparison graph of the CV curves at different bending angles, *i.e.*, 0°, 45° and 90°. (e) *C*_sp_*vs.* bending angle graph of the Co-MOF pouch cell.

The pouch cell GCD behaviour was analyzed over *I*_d_ varied from 0.25 A g^−1^ to 0.87 A g^−1^ in the optimized voltage window, as displayed in Fig. 13(d) and (e). The GCD curves showed a capacitive profile with reversible charge–discharge behaviour and the corresponding *C*_sp_ values derived from the curves are shown in Fig. 13(e). The notable *iR* drop in the discharge curve originated from the internal resistance of the device. This included the intrinsic low electrical conductivity of the Co-MOF electrode, which restricted efficient electron transport, along with contact resistance between the active material and current collector. Ion diffusion limitations within the porous MOF structure and incomplete electrolyte penetration further led to increased resistance and polarization effects. The higher mass loading, electrode thickness and possible imperfect interfacial contact of the pouch cell configuration also contributed to the overall resistive losses. The highest *C*_sp_ of 1.68 F g^−1^ was measured at an *I*_d_ of 0.25 A g^−1^ while stable capacitive behaviour was sustained, suggesting good rate capability as shown in Fig. 13(f). This significant difference in *C*_sp_ between the three-electrode setup and the symmetric device was due to the fundamental differences in measurement conditions. The three-electrode setup demonstrated the inherent characteristics of an individual electrode under ideal conditions, with minimal influence from the counter and reference electrodes. However, the symmetric device was limited by combined electrode effects, internal resistance and reduced active material utilization. As a result, the device exhibited significantly lower capacitance values. The Co-MOF pouch cell was further evaluated by calculating its *E*_d_ and *P*_d_ using Ragone analysis, as indicated in Fig. 13(g). A maximum *E*_d_ of 0.22 W h kg^−1^, accompanied by *P*_d_ of 43.75 W kg^−1^, was achieved by the pouch cell. The Co-MOF pouch cell was tested over 10 000 repeated charge–discharge cycles with the corresponding coulombic efficiency to determine the operational stability of the device. The device delivered superior cycling stability, maintaining approximately 41.2% of its original capacitance after 10 000 repeated charge–discharge cycles with a coulombic efficiency of 87%, as depicted in Fig. 13(h). Table S1 shows the comparison of the electrochemical behaviour of the Swagelok and the pouch cell device. To assess its practical applicability, the pouch cell was further employed to power a red LED. Although successful illumination was attained, the device maintained emission for merely 25–30 seconds. Therefore, upon comparing the two setups, the pouch cell exhibited a significantly lower *C*_sp_ than the Swagelok cell. This difference was mainly due to the variations in the electrode–electrolyte interface. The liquid electrolyte in the Swagelok cell ensured better ionic conductivity and effective penetration of ions into the Co-MOF structure, which enabled improved charge transfer. Also, the gel electrolyte used in the pouch cell showed comparatively lower ionic mobility and limited electrode wetting, which led to higher internal resistance and restricted ion transport. As a result, the decreased accessibility of active sites and slower electrochemical reactions led to the reduced efficiency of the pouch cell. Despite this, these findings highlight the significance of the Co-MOF electrodes for supercapacitor applications, owing to their excellent electrochemical behaviour and enduring stability. The mechanical flexibility of the symmetric cell configuration was assessed through the observation of CV profiles at various bending angles of 0°, 45° and 90°, as illustrated in Fig. 14(a)–(c). The CV curves recorded at 200 mV s^−1^, maintained their overall features and shape without significant distortion, thereby demonstrating exceptional electrochemical stability at different bending angles. Furthermore, the *C*_sp_ increased from 3.17 F g^−1^ at 0° to 5.64 F g^−1^ at 45° and subsequently to 11.35 F g^−1^ at 90°, as presented in Table S2, indicating enhanced charge storage performance upon bending, as demonstrated in Fig. 14(d) and (e). The obtained results confirm the robust mechanical stability and efficient electrochemical performance of the device, thus highlighting its promise for application in devices, specifically in flexible and wearable electronics.

## 4. Conclusion

Herein, a Co-MOF was synthesized through a hydrothermal route and then integrated onto nickel foam to examine its electrochemical properties for supercapacitor applications. Structural analysis using XRD verified the synthesis of a highly crystalline and well-structured Co-MOF, with specific diffraction peaks corresponding to its characteristic crystal planes. Prominent peaks at 17.49° and 18.70° align with the standard diffraction pattern of the Co-MOF, indicating that it exhibits high crystallinity and phase purity. Morphological analysis *via* FE-SEM demonstrated irregularly shaped, spherical grains exhibiting a rod-like structure. XPS revealed the coexistence of Co^2+^ and Co^3+^ oxidation states, which is advantageous for redox-active charge storage. The electrochemical behaviour of the Co-MOF electrode was comprehensively assessed through CV, GCD, and EIS in KOH and NaOH electrolytes. Among the investigated electrolytes, 1 M NaOH emerged as the most effective medium, exhibiting superior electrochemical behaviour relative to KOH and higher NaOH concentrations. The highest *C*_sp_ of 1147.2 F g^−1^ was achieved by the Co-MOF at 0.5 A g^−1^ during GCD and 852.5 F g^−1^ at a sweep rate of 2 mV s^−1^ during CV in 1 M NaOH. This was followed by 3 M and 5 M NaOH, which produced *C*_sp_ values of 714 F g^−1^ and 677 F g^−1^, respectively, highlighting the importance of optimized electrolyte concentration. Cycling stability measurements demonstrated excellent durability with approximately 98% coulombic efficiency even after 10 000 cycles in 1 M NaOH. EIS further confirmed favourable kinetics in the electrolyte, as demonstrated by the lowest *R*_s_ of 3.05 Ω and improved ion transport performance. Furthermore, a symmetric supercapacitor device assembled with the Co-MOF electrode and 1 M NaOH electrolyte exhibited a *C*_sp_ value of 37.7 F g^−1^ from CV measurements and 14.9 F g^−1^ based on GCD measurements. An *E*_d_ of 3.73 W h kg^−1^ was recorded for the assembled device with a corresponding *P*_d_ of 118.75 W kg^−1^, maintaining 98.66% coulombic efficiency with 43.96% retention after 10 000 repeated cycling tests. Similarly, the Co-MOF pouch cell demonstrated a *C*_sp_ of 21.42 F g^−1^ at 2 mV s^−1^ for CV and a maximum of 1.68 F g^−1^ when evaluated at 0.25 A g^−1^ for GCD. Ragone analysis indicated an *E*_d_ value of 0.22 W h kg^−1^ at a corresponding *P*_d_ of 43.75 W kg^−1^. The device also maintained about 87% coulombic efficiency with 41.2% retention after undergoing 10 000 continuous cycling tests. Overall, the observations indicate that electrolyte preference and concentration are key factors in determining the overall electrochemical behaviour of Co-MOF electrodes, highlighting their prospects for efficient energy storage devices.

## Author contributions

Mrinalini Sharma: writing – original draft, review, editing, investigation, analysis, and data curation. Manas Nasit: formal analysis, investigation, and data curation. Nitin Kumar Gautam: data curation, formal analysis. Shruti Lavania: investigation, formal analysis. Saurabh Dalela: investigation and formal analysis. P. A. Alvi: investigation and formal analysis. Nagih M. Shalaan: formal analysis. Ranjeet Kumar Brajpuriya: review, editing, investigation, formal analysis, and resources. Aditya Sharma: investigation and formal analysis. Shalendra Kumar: supervision, editing, reviewing, conceptualization, and data curation.

## Conflicts of interest

The authors declare that they do not have any known competing financial interests or personal relationships that could have appeared to influence the work reported in this paper.

## Supplementary Material

RA-016-D6RA01795A-s001

## Data Availability

The data used to support the findings of this study are included in the article. The raw data that support the findings of this study are available from the corresponding author upon reasonable request. Supplementary information (SI) is available. See DOI: https://doi.org/10.1039/d6ra01795a.
