# Supplementary material for: Electrolyte-driven modulation of charge storage mechanisms in Co metal–organic frameworks for advanced supercapacitors
Source: RSC Adv. 2026 Apr 15;16(22):19851–69. doi: 10.1039/d6ra01795a (PMC13080442; doi:10.1039/d6ra01795a)
Supplement: RA-016-D6RA01795A-s001 [file RA-016-D6RA01795A-s001.pdf]

Supplementary Information (SI) for RSC Advances.
This journal is © The Royal Society of Chemistry 2026

Supplementary Information
Electrolyte-Driven Modulation of Charge Storage Mechanisms in Co Metal–Organic Frameworks for Advanced Supercapacitors

Mrinalini Sharma[1], Manas Nasit[1], Nitin Kumar Gautam[1], Shruti Lavania[1], Saurabh Dalela[2], P.A Alvi[3], Nagih M. Shalaan[4], Ranjeet Kumar Brajpuriya[1†], Aditya Sharma[1], Shalendra Kumar[1*]

[1]Department of Physics, School of Advanced Engineering, UPES, Dehradun 248007, India

[2]Department of Pure & Applied Physics, University of Kota, Kota, Rajasthan, 324005, India

[3]Department of Physical Science, Banasthali Vidyapith, Banasthali, Rajasthan, 304022, India

[4]Department of Physics, College of Science, King Faisal University, P.O. Box 400, Al-Ahsa 31982, Saudi Arabia

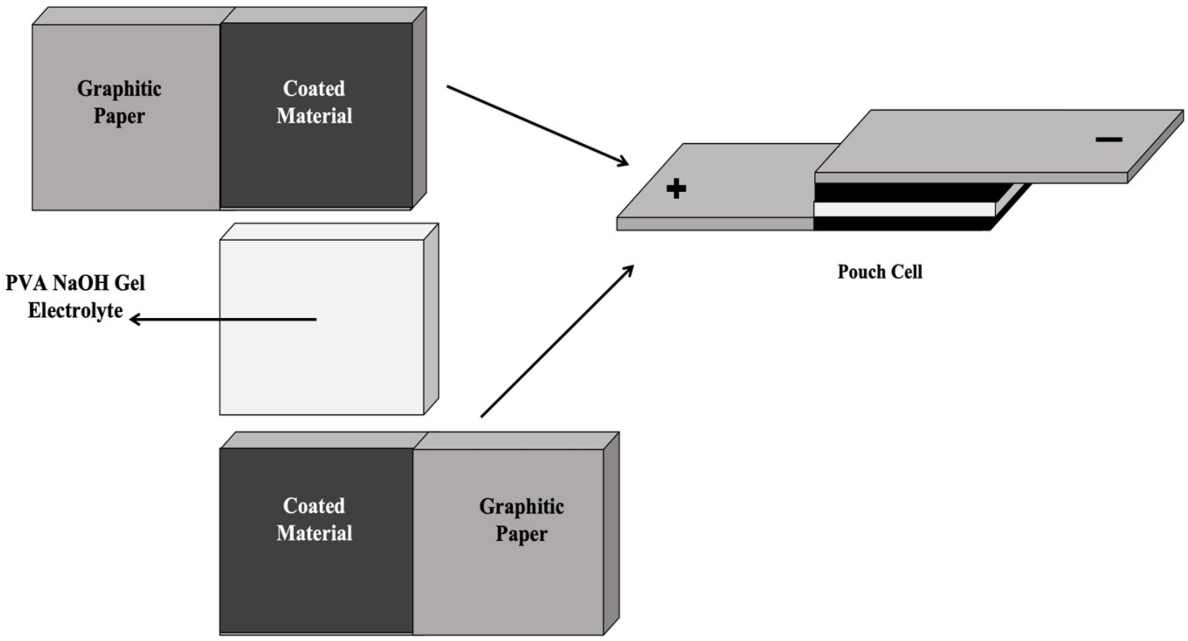

Figure. S1. Schematic illustration of Co-MOF Pouch Cell.

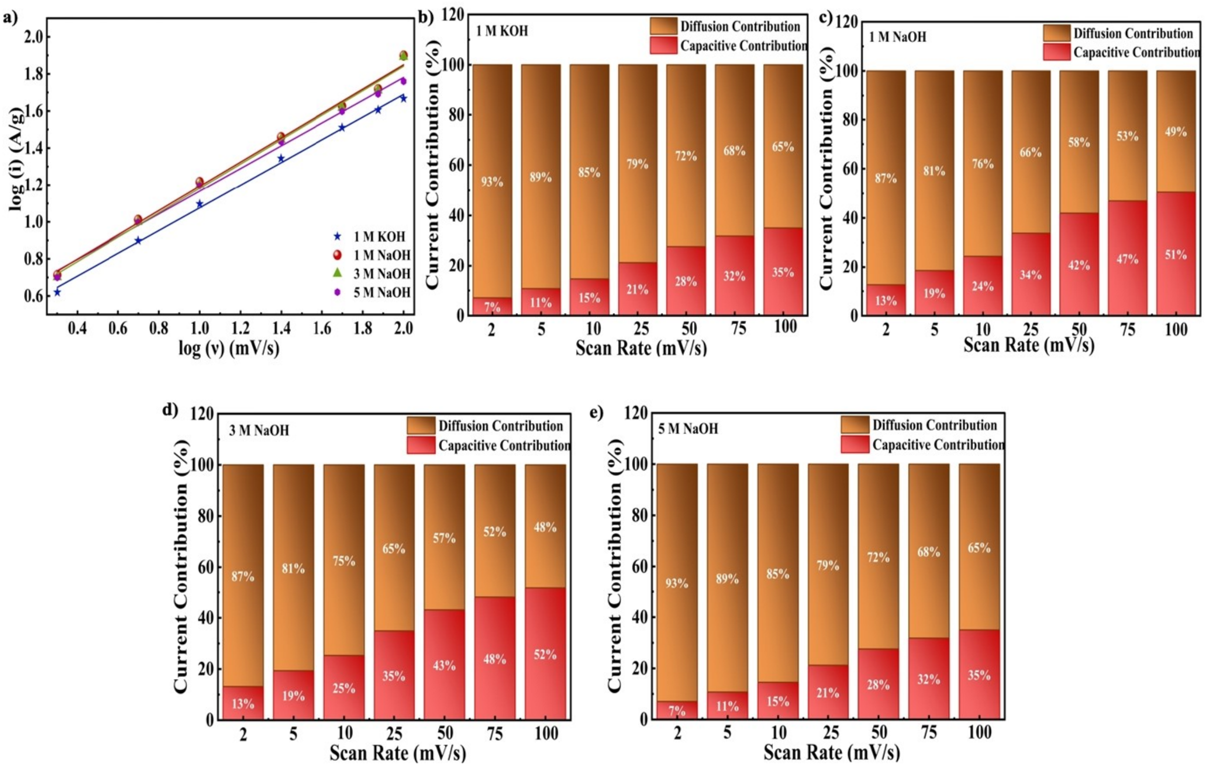

Fig. S2 (a) Log (i) vs Log (v) Analysis of Co-MOF in 1M KOH and 1M NaOH electrolytes at different molarities (3M NaOH and 5M NaOH), Analysis of Surface-controlled and Diffusion-controlled charge storage in Co-MOF for **(b)** 1M KOH **(c)** 1M NaOH **(d)** 3M NaOH **(d)** 5M NaOH

Table:

Table S1. Comparison of Electrochemical behaviour of Swagelok & Pouch cell using CV, GCD and Capacitance retention (%).

Device	CV (at 2mV/s)	GCD (at 0.25 A/g)	Retention (%)
**Swagelok**	37.7 F/g	14.9 F/g	43.96%
**Pouch Cell**	21.42	1.68 F/g	41.2 %

Table S2. Influence of bending angle on the C_{sp} of the flexible device.

**Bending Angle(°)**	**Specific Capacitance(F/g)**
0	3.17
45	5.64
90	11.35
